# Palatal canine impaction is not associated with third molar agenesis

**DOI:** 10.1093/ejo/cjaf008

**Published:** 2025-03-05

**Authors:** Christianna I Papadopoulou, Maria Athanasiou, Nikolaos Gkantidis, Georgios Kanavakis

**Affiliations:** Department of Orthodontics and Pediatric Dentistry, UZB-University School of Dental Medicine, University of Basel, Mattenstrasse 40, CH-4058 Basel, Switzerland; Department of Orthodontics and Dentofacial Orthopedics, Center for Oral Health Sciences CC3, Charité-Universitätsmedizin Berlin, Assmannshauser Straße 4-6, DE-14197, Berlin, Germany; Department of Orthodontics and Pediatric Dentistry, UZB-University School of Dental Medicine, University of Basel, Mattenstrasse 40, CH-4058 Basel, Switzerland; Department of Orthodontics and Dentofacial Orthopedics, School of Dental Medicine, University of Bern, Freiburgstrasse 7, CH-3010 Bern, Switzerland; Department of Orthodontics and Pediatric Dentistry, UZB-University School of Dental Medicine, University of Basel, Mattenstrasse 40, CH-4058 Basel, Switzerland; Department of Orthodontics, School of Dentistry, National and Kapodistrian University of Athens, Thivon 2, GR-11527, Athens, Greece

**Keywords:** palatally impacted canines, palatal canine impaction, palatally displaced canines, third molar agenesis, tooth agenesis

## Abstract

**Background/Objectives:**

Third molar agenesis and palatally impacted canines (PICs) are two independent dental phenotypes with different developmental backgrounds. Isolated reports indicate a common genetic origin for both, however, current data is inconsistent. The aim of this study was to investigate the presence of third molar agenesis in individuals with PICs, compared to individuals without PICs.

**Materials/Methods:**

This retrospective case-control study comprised 310 individuals (188 females and 122 males), half of whom presented with unilateral or bilateral PICs. Individuals with other dental anomalies of known genetic origin were excluded. The association between PICs and third molar agenesis was assessed using four regression models, with PIC as the dependent variable and sex, age, and third molar agenesis as predictors. One model treated PIC as a nominal variable (pattern) and the other as ordinal (severity), and both were run testing either third molar agenesis severity or third molar agenesis patterns. All statistical tests were performed assuming a type-1 error of 5%.

**Results:**

There was no significant association between canine impaction and third molar agenesis in any of the four regression models. Neither the severity nor the patterns of palatally impacted canines were associated with either the severity or the patterns of third molar agenesis (*P* > .05).

**Limitations:**

Due to the common racial background of all participants, the results of this investigation might not be generalizable to the general population.

**Conclusions/Implications:**

Palatal canine impaction is not associated to third molar agenesis, after accounting for age, sex, and various patterns of PICs and third molar agenesis. These results indicate that these two dental phenotypes do not share a common biological mechanism for their occurrence.

## Introduction

Tooth eruption is a physiologic mechanism during which a tooth migrates from its developmental position, through the periodontal tissues, into the oral cavity to assume its functional position in the dental arch [1]. Systemic or local factors might interrupt this process and obstruct the eruption of a tooth, leading to a pathological situation named tooth impaction. It presents a relatively frequent challenge in orthodontic clinical practice, often recognized by chance during a routine examination.

The permanent maxillary canine is the second most frequently impacted tooth, after the third molar, with a prevalence of 1%–3% in European populations [2]. Epidemiological data show that palatally impacted canines (PICs) are more common than buccally impacted canines (BICs) with a 2:1 ratio, there are twice as many females than males with PICs, and a bilateral occurrence ranges between 19% and 45% [3]. The etiology behind maxillary canine impaction is a topic of ongoing discussion. BICs have often been correlated with maxillary crowding, whereas 82%–85% of PICs occur in patients without crowding [4, 5]. This is the reason that PICs are thought to have a genetic background. There are two main theories associated with palatal canine impaction, the guidance theory and the genetic theory. The guidance theory suggests that the eruption of the maxillary canine is influenced by local factors, such as the mesiodistal crown width, the root length, and the malformation of the maxillary lateral incisor, or the retention or absence of the primary canine [6–9]. The genetic theory proposes that canine impaction is linked to genetic factors. This theory is supported by studies that have linked PICs with other dental anomalies of genetic origin, such as missing or peg shaped lateral incisors, other missing teeth, tooth transposition, teeth spacing and late developing dentitions [3, 4, 6, 10–12]. These research findings suggest a hereditary component, which supports the genetic theory as the leading cause for PICs. A systematic review of the available evidence regarding the two theories failed to confirm one over the other. However, taking into consideration the sampling and methodology of the included studies, it appears that the guidance theory is less robust in explaining the displacement of the maxillary canine germ [13].

The association between PICs and tooth agenesis has been shown in several studies and pertains particularly to the agenesis of lateral incisors [4], premolars [14] and third molars [10, 15]. Tooth agenesis is a common dental anomaly, occurring in 6.4% of the population, although there are differences among various ethnicities and populations [16, 17]. The most commonly missing permanent teeth are the second mandibular premolars and the maxillary lateral incisors [18], not accounting for third molars, which are missing much more frequently, in more than 20% of the population [19]. Tooth agenesis is a genetic dental anomaly, found in isolation or as part of a syndrome [20]. Isolated tooth agenesis is familial and is inherited in an autosomal-dominant manner [21]. A study on a family group with missing second premolars and third molars identified a point mutation in the MSX1 gene [22]. Similarly, a mutation in the PAX9 transcription factor has been linked to familial tooth agenesis, also affecting third molars [23]. Other associated genes include AXIN2, EDA, IRF6, FGFR1, and WNT10A [24, 25]. Previous research suggested a common genetic basis for several processes of dental and craniofacial development [26]. Tooth agenesis, in particular, has been associated to reduced craniofacial dimensions and a more brachyfacial skeletal pattern with shorter anterior facial height and a more concave profile [27–30]. Also, craniofacial shape differences were recently identified between individuals with palatal canine impaction and normal controls, providing important information regarding the developmental origin of PICs [31].

This existing evidence raises the question whether isolated third molar agenesis and palatal canine impaction are developmentally linked, since both phenotypes are associated with specific craniofacial morphologies. This link has been studied before, however, confounding factors of genetic origin were not controlled [10, 15]. Therefore, the aim of this investigation was to study the occurrence of third molars agenesis in individuals with isolated palatal canine impaction compared to normal controls. The results will disseminate significant information regarding the developmental relationship between these two frequent dental phenotypes and enrich the literature about the etiology of positional anomalies of the maxillary canine.

## Materials and methods

### Ethical approval

The study proposal received joint approval by the Ethics Committee of Northwest and Central Switzerland (EKNZ) and the Ethic Committee of the Canton of Bern Switzerland (Project Nr: 2021-00311).

### Sample

Orthodontic patient records were collected from a large data base of the following orthodontic clinics: (a) University of Basel, Switzerland, (b) University of Bern, Switzerland, in accordance with the following criteria:

(i) Confirmed presence of palatal impacted canines (PICs) (unilateral or bilateral). The presence of palatal canine impaction was confirmed through the subjects’ treatment history and/or radiographically (with a Cone Beam CT (CBCT) scan) and/or through the presence of palatal canine bulge on the pre-treatment models. If the diagnosis was unclear, the records were evaluated with the senior author and if agreement was still not reached the subject was excluded from the investigation
(ii) No craniofacial malformations, syndromes, systemic diseases, or any other anomalies affecting craniofacial morphology, as reported in the subjects’ medical record.
(iii) Individuals older than 9 years of age at the time of pre-treatment cephalometric radiograph. In cases where the eligible pre-treatment cephalometric radiograph was obtained at an age younger than 13 years of age, records that confirmed the palatal impaction until a minimum age of 13 years old should have been available. No treatment that could have affected the canine position between 9 and 13 years of age should have been performed.
(iv) Pre-treatment lateral cephalometric radiograph of adequate diagnostic quality in maximal intercuspation, with a reference ruler at the mid-sagittal level.
(v) Panoramic radiographs of diagnostic quality.
(vi) No history of interventions known to influence craniofacial morphology, such as orthodontic treatment.
(vii) No history of canine extraction.
(viii) Total absence of any other dental anomalies in the tooth number, size, or form of any tooth, except for third molars.

The data base was examined in accordance with the aforementioned criteria for the collection of the study and control groups and each subject was reviewed for their medical and dental history, as well as for their panoramic, cephalometric and/or peri-apical radiographs. Individuals with tooth agenesis, apart from third molars, or any other dental anomalies were excluded in order to not introduce confounders that could affect the primary outcome. The data were recorded by using Microsoft Excel and were classified according to the identification of the impacted canines. Based on the above inclusion criteria, the final sample for this investigation comprised 310 individuals (188 females and 122 males), half of which were presented with unilateral or bilateral palatal impaction of maxillary canines (study group). The study group was matched for sex and age to the control group to account for sex dimorphism in craniofacial morphology and for the effect of growth on the craniofacial structures. Therefore, for every subject in the study group (N = 155) there was a subject in the control group (N = 155) of the same age and sex.

To assess whether the final sample was adequate to investigate the primary outcome we applied a post-hoc sample size calculation indicated for ordinal regression models, according to Peduzzi et al [32]. In this study there were 155 cases of palatal canine impaction (events) and three independent variables (sex, age, number of third molars) that could predict canine impaction. So, the ratio EPV (Events Per Variable) was 155/3 = 51.67, which is considered to be excellent and confirms the robustness of the present sample population.

### Methodology

The presence of third molars was assessed by a single, blinded examiner, who was not aware of the purpose of the study nor about the status (impacted/non impacted) of the permanent canines. The examiner assessed all 310 panoramic x-rays and noted the presence of third molars by inserting a binary variable in an Excel sheet for every third molar (0: absent, 1: present). In addition, demographic information for every individual were also recorded, namely sex, date of birth, date of record acquisition and congenital absence of teeth.

The patterns of third molar agenesis in the sample were recorded using a modification of the TAC system [20, 33]. This system assigns a binary value for each tooth in a dentition, indicating its presence or absence. Thus, it provides a unique identification number for each individual, representing a specific tooth agenesis pattern. Here, the only possibly missing teeth were teeth 18, 28, 38, 48. This means that there are 2 [4]=16 possible combinations of present or absent third molars. Unique identifiers for each missing third molar, displayed in Table 1, were selected instead of using binary values, so that if an individual was missing teeth 18 and 48, they would be assigned the unique code 1 + 8 = 9. This allowed for the unique coding of all patterns in missing third molars through a single value enabling the investigation of associations of these patterns to the presence of palatal impacted canines.

**Table 1.** Identifiers for each missing third molar, based on the modified TAC system.

Unique identifiers for each missing third molar				
Third molar missing	18	28	38	48
Assigned identifier	1	2	4	8

In order to allow for a more comprehensive evaluation of the data, PICs were also recorded based on their laterality, as a nominal variable as follows: 0 = No PIC, 1 = Right PIC, 2 = Left PIC, 3 = Bilateral PIC. Assuming that a bilateral canine impaction is a more severe phenotype than a unilateral canine impaction, PICs were also recorded as an ordinal variable as follows: 0 = No PIC, 1 = Unilateral PIC, 2 = Bilateral PIC. This categorization increased the robustness of the statistical analyses as described below.

### Statistical analysis

The statistical analysis was designed in order to assess the predictive value of third molar agenesis for the presence of palatal canine impaction, when controlling for sex and age.

Two statistical models were developed, an ordinal regression model and a nominal regression model with ‘canine impaction’ as dependent variable and ‘third molar agenesis’, ‘sex’ and ‘age’ as the predictors. The former model assumed that the severity of palatal canine impaction increases as the phenotype becomes bilateral (ordinal model), while the latter assumed that the phenotype is expressed in four different categories, independent from each other (nominal model). In each of the approaches, ‘third molar agenesis’ was examined in two different ways. First, specific patterns of missing third molars were not taken into consideration and the variable was expressed as the number of present third molars (0, 1, 2, 3, or 4). Then, the same analyses were performed using the TAC code for each pattern of third molars agenesis in order to tests whether any association was present with specific patterns of missing third molars. This resulted in a total of four regression models. Model fitting information was determined using the Chi-Square value and the strength of the effect was assessed with the Pseudo-R^2^ value, through the McFadden test. A type-1 error of 5% was assumed in all statistical analyses.

In a sub analysis, the presence of palatally impacted canines was also regressed against the presence of maxillary third molars in individuals who had both mandibular third molars present. This was done in order to also test the assumption that the presence of palatal canine impaction is associated to the presence of maxillary third molars, potentially sharing a common biological background related to the development of the maxilla.

### Method error

The error of the method was tested through repetition of the data generation by the same examiner after a wash-out period of 3 weeks. The Cohen’s Kappa coefficient was used to assess intra-examiner reliability.

## Results

### Method error

The method error assessment showed a perfect agreement within the operator who performed the data generation. Cohen’s Kappa value was 1.

### Patterns of palatal canine impaction and third molar agenesis

The patterns presented for third molar agenesis and palatal canine impaction are presented in Tables 2 and 3. According to the study design, half of the individuals presented palatal canine impaction. Most of those cases presented with unilateral canine impaction (80% of PIC group) (Table 2).

**Table 2.** Patterns of third molars agenesis. Grey cells indicate an absent tooth and white cells a present tooth.

18	28	38	48	N	Percent (%)	TAC-code
				222	71.6	0
				21	6.8	15
				15	4.8	12
				13	4.2	3
				9	2.9	4
				6	1.9	1
				6	1.9	2
				5	1.6	8
				3	1	11
				2	0.6	6
				2	0.6	7
				2	0.6	13
				2	0.6	14
				1	0.3	10
				1	0.3	5
Total				310	100	

**Table 3.** Patterns of palatal canine impaction. Grey cells indicate the presence of a palatally impacted canine and white cells the presence of a normal canine.

13	23	N	Percent (%)
		155	50
		65	21
		59	19
		31	10

In regard to third molar agenesis, most individuals had all third molars present (71.6%). The most commonly occurring pattern for third molar agenesis was absence of all third molars (6.8%), followed by the absence of only mandibular third molars (4.8%), and absence of only maxillary third molars (4.2%) (Table 3).

### Association between PIC and third molar agenesis

**Figure 1.**
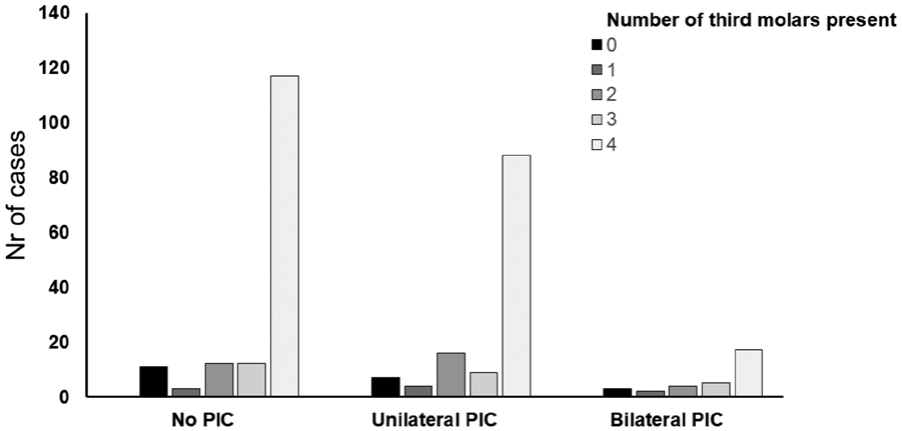
Bar chart showing the association between number of third molars present and severity of palatal canine impaction (PIC).

The statistical analyses revealed no significant association between the agenesis of third molars and palatal canine impaction. This was evident in all regression models that were applied on the data (Table 4). With the assumption that palatal canine impaction is a phenotype the severity of which increases as it presents unilaterally or bilaterally, the predictive value of the model was 0.4% (McFadden pseudo R^2^ = 0.004) and the association between third molar agenesis severity and palatal canine impaction showed no statistical significance (χ^2^ = 19.13, *P* = .328) (Table 4, Fig. 1). Furthermore, no third molar agenesis pattern was associated with palatal canine impaction severity (Table 4, Supplementary Table 1).

**Table 4.** Regression of palatal canine impaction (PIC) over sex, age and third molar agenesis.

	Chi-Square	Mc Fadden pseudo R^2^	*P*-value
Model 1 Dependent variable: PIC ordinal Predictors: Sex, Age, Number of present third molars	19.130	0.004	.328
Model 2 Dependent variable: PIC nominal Predictors: Sex, Age, Number of present third molars	5.090	0.007	.532
Model 3 Dependent variable: PIC ordinal Predictors: Sex, Age, Third molar agenesis pattern	11.647	0.020	.768
Model 4 Dependent variable: PIC nominal Predictors: Sex, Age, Third molar agenesis pattern	50.802	0.067	.364
Model 5 Dependent variable: PIC ordinal Predictors: Sex, Age, Number of present maxillary third molars	5.925	0.013	.205
Model 6 Dependent variable: PDC nominal Predictors: Sex, Age, Number of present maxillary third molars	10.831	0.018	.543

In order to explore if the laterality of palatal canine impaction was related to third molar agenesis severity, the main hypothesis was also tested under the assumption that palatal canine impaction is a phenotype with four distinct presentations, namely ‘no impaction’, ‘left palatal impaction’, ‘right palatal impaction’ and ‘bilateral impaction’. However, no significant associations were revealed under this hypothesis testing either (χ^2^ = 5.09, *P* = .532, McFadden pseudo R^2^ = 0.007) (Table 4). Palatal canine impaction was also not associated with third molar agenesis patterns, when tested as a nominal variable (Table 4).

**Figure 2.**
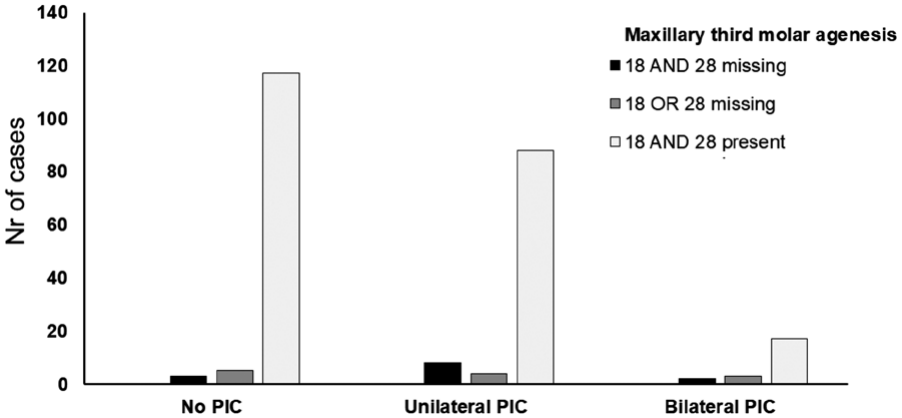
Bar chart showing the association between number of maxillary third molars present and severity of palatal canine impaction (PIC).

The results of the sub analysis did not reveal any significant results either. There was no association between palatal canine impaction and maxillary third molar agenesis in individuals with both mandibular third molar present (χ^2^ = 5.925, *P* = .205, McFadden pseudo R^2^ = 0.013) (Table 4, Fig. 2).

## Discussion

The premise for this study was to explore the association between palatally impacted canines and third molar agenesis, based on the assumption that the two phenotypes might share a common genetic background [3, 10, 11]. Existing evidence shows that palatal canine impaction often occurs in patients with tooth agenesis [11]. Based on this observation, and if a genetic background for palatal canine impaction is considered plausible, it can be speculated that there are common genetic signals influencing the development of the third molar and the eruption of the palatal canine. Nevertheless, the results of our study showed no significant association between palatal impacted canines and third molar agenesis, suggesting that these two phenotypes are independent of one another.

The genetic basis of tooth agenesis is well-established, with several genes identified as controlling factors, namely MSX1, PAX9, AXIN2, EDA, IRF6, FGFR1, and WNT10A [22, 34, 35]. These genes are involved in dental development; therefore, their mutations can lead to the congenital absence of teeth [35, 36]. Previous studies that have demonstrated associations between palatal impacted canines and the agenesis of other teeth, such as lateral incisors, premolars, and third molars [6, 7, 10, 11, 15] support the theory that palatal canine impaction is often accompanied by other dental anomalies, thereby implying a shared genetic or developmental background. Vastardis et al. identified a defective MSX1 gene, showing its important role in the agenesis of third molars and mandibular second premolars. This finding suggested that MSX1 is a likely candidate gene for controlling the development of the posterior (molar) region [22, 34]. Similarly, PAX9, which has been associated with the agenesis of all molars, is recognized for its significance in posterior orofacial development [37]. Neubüser et al. linked the PAX9 transcription factor to the positioning of tooth buds at the mesenchymal level, a connection that may be important for understanding the genetic basis of tooth displacement anomalies such as palatal canine impaction [38]. In addition, Saito et al. (2006) and Devi et al. (2019) showed that genetic polymorphisms in the MSX1 and PAX9 genes might be associated to a genetic predisposition to palatal canine impaction, although both studies evaluated a relatively small sample population [39, 40].

Although some reports tend to agree with the results of this study [41], our findings diverge from the vast majority of previous studies in this field. We attribute this to a crucial difference in sample selection as most of the previous studies did not exclude individuals with other missing teeth or other genetic dental anomalies, such as microdontia or tooth transpositions [3, 10, 11, 15, 39, 42–44]. Such individuals are known to be more likely to present third molar agenesis, thus introducing significant bias in the results [45]. After controlling for these factors, no association between palatal canine impaction and third molar agenesis was demonstrated, indicating that these two phenotypes occur independently from each other.

Furthermore, the statistical analyses were conducted in two ways to assess all possible phenotypic variations of palatal canine impaction. Unilateral and bilateral palatal canine impaction were examined as separate phenotypes (nominal model) and as the same presentation of the same phenotype, with different severity levels (ordinal model). This dual-model approach provided a comprehensive framework for testing our hypothesis from multiple angles, enhancing the robustness of the statistical analyses. In both models, no significant association between palatal canine impaction and third molar agenesis was identified. We also explored the possibility of an association between palatal canine impaction and isolated maxillary third molar agenesis, speculating that a developmental disturbance of the maxilla could theoretically be a local factor affecting both phenotypes. Being the last tooth of the human dentition, the third molar presents a higher chance for disturbed eruption pattern [19]. Thus, in the case of a developmental discrepancy in the maxilla, leading to palatal canine impaction, agenesis of the third molar could be more likely. Nevertheless, this assumption was not confirmed either from our results.

Admittedly, our findings were unexpected, since at the stage of conceptualization it was assumed that an association would be evident. Previous investigations have indeed reported that third molar agenesis occurs more frequently in the presence of palatal canine impaction. However, methodological concerns regarding the confounding effect of other genetically controlled phenotypes, such as small or missing lateral incisors raise questions about the reliability of their findings [15]. Recently published data have also strengthened the theory of a genetic origin for PICs, revealing significant craniofacial shape differences between individuals with palatal canine impaction and normal controls [31]. The former present with a shorter anterior height of the lower face and a less convex profile. The same craniofacial form has been observed in individuals with third molar agenesis or agenesis of other permanent teeth [29]. Nevertheless, despite these indications of an existing association, this was not found in the present study. A possible biological explanation for these findings is that palatal canine impaction and third molar agenesis are two genetically regulated phenotypes, both linked to overall craniofacial development, but they arise through distinct underlying mechanisms.

The results of this study provide further information to the ongoing discussion about the etiology of PICs. From a clinical perspective, our results suggest that practitioners should not assume a direct link between palatal canine impaction and third molar agenesis during orthodontic diagnosis and treatment planning. Palatal canine impaction should be evaluated as an isolated situation, concerning third molar formation. Our findings remain to be tested further by future genetic research investigating the origin of palatal canine impaction.

### Limitations

Due to the common racial background of all participants, the results of this investigation might not be generalizable to the general population. Also, there might be other local or systemic factors that could have had an effect on the results, which are either not well established or fall within the spectrum of normal phenotypic variation. The effect of such factors is controlled by the large sample size and by the strict matching between the study and the control group.

## Conclusions

There is no association between palatal canine impaction patterns and severity with either third molar agenesis patterns or severity. This finding adds important information to existing literature and provides strong indication that these two genetically controlled phenotypes are the result of different developmental pathways.

## Supplementary Material

cjaf008_suppl_Supplementary_Tables_1

## Data Availability

All raw data are publicly available by Zenodo open depository, under the following link:10.5281/zenodo.13996771. This excludes identifying information of study participants.
