# Supplementary material for: Palatal canine impaction is not associated with third molar agenesis
Source: Eur J Orthod. 2025 Mar 5;47(2):cjaf008. doi: 10.1093/ejo/cjaf008 (PMC11880766; doi:10.1093/ejo/cjaf008)
Supplement: cjaf008_suppl_Supplementary_Tables_1 [file cjaf008_suppl_supplementary_tables_1.docx]

Supplementary table 1. Effect of third molar agenesis patterns (TAC scores) on the presence of palatal canine impaction.

| Dependent variable:  Palatal canine impaction (ordinal data) |  |  |  |  |
| --- | --- | --- | --- | --- |
|  | TAC-code | N | Estimate | P-value |
|  | 0 | 222 | -0.142 | 0.753 |
|  | 1 | 6 | 1.117 | 0.210 |
|  | 2 | 6 | 0.127 | 0.888 |
|  | 3 | 13 | 0.926 | 0.171 |
|  | 4 | 9 | 0.199 | 0.796 |
|  | 5 | 1 | 1.114 | 0.569 |
|  | 6 | 2 | 1.135 | 0.426 |
|  | 7 | 2 | -0.178 | 0.903 |
|  | 8 | 5 | -0.181 | 0.853 |
|  | 9 | 0 | N/A | N/A |
|  | 10 | 1 | 1.119 | 0.566 |
|  | 11 | 3 | 1.936 | 0.103 |
|  | 12 | 15 | -0.179 | 0.787 |
|  | 13 | 2 | 1.146 | 0.421 |
|  | 14 | 2 | -0.174 | 0.904 |
|  | 15 | 21 | 0.000 | 1.000 |
